# Football player dominant region determined by a novel model based on instantaneous kinematics variables

**DOI:** 10.1038/s41598-021-97537-4

**Published:** 2021-09-14

**Authors:** Fabio Giuliano Caetano, Sylvio Barbon Junior, Ricardo da Silva Torres, Sergio Augusto Cunha, Paulo Régis Caron Ruffino, Luiz Eduardo Barreto Martins, Felipe Arruda Moura

**Affiliations:** 1 Laboratory of Applied Biomechanics, Department of Sport Sciences, Centre of Physical Education and Sport, State University of Londrina, Km 380 Celso Garcia Cid Road, University Campus, Londrina, Parana 86057-970 Brazil; 2 Department of Computer Science, State University of Londrina, Londrina, Brazil; 3 Department of ICT and Natural Sciences, Norwegian University of Science and Technology (NTNU), Ålesund, Norway; 4 Department of Sport Sciences, University of Campinas, Campinas, Brazil; 5 Department of Mathematics, University of Campinas, Campinas, Brazil

**Keywords:** Mathematics and computing, Applied mathematics, Computational science

## Abstract

Dominant regions are defined as regions of the pitch where a player can reach before any other and are commonly determined without considering the free-spaces in the pitch. We presented an approach to football players’ dominant regions analysis, based on movement models created from players’ positions, displacement, velocity, and acceleration vectors. 109 Brazilian male professional football players were analysed during official matches, computing over 15 million positional data obtained by video-based tracking system. Movement models were created based on players’ instantaneous vectorial kinematics variables, then probabilities models and dominant regions were determined. Accuracy in determining dominant regions by the proposed model was tested for different time-lag windows. We calculated the areas of dominant, free-spaces, and Voronoi regions. Mean correct predictions of dominant region were 96.56%, 88.64%, and 72.31% for one, two, and three seconds, respectively. Dominant regions areas were lower than the ones computed by Voronoi, with median values of 73 and 171 m^2^, respectively. A median value of 5537 m^2^ was presented for free-space regions, representing a large part of the pitch. The proposed movement model proved to be more realistic, representing the match dynamics and can be a useful method to evaluate the players’ tactical behaviours during matches.

## Introduction

Football is a team sport characterised as a sociomotor physical activity that involves cooperative (with teammates) and competitive (with opponents) interactions, as well as, environmental uncertainties^1^. These relationships among the players show a behaviour previously reported to animals on the ecological approach^2^ applied in team sports to understand team synergies^3^, in which they perceive the environment around them and adapt accordingly. The environmental constraints^4^ in the case of football, for example, are the teammates or opponent players, the pitch, and the ball. Thus, players experience a perception–action process in which they make decisions based on the actions of their neighbour players, as well as their actions also affect the neighbour players^5,6^.

The interactions among neighbouring players are a relevant concept to evaluate the use of space by the teams and competition for the pitch regions because it has been used to compute the players’ dominant regions^7^. The player dominant region is defined as the pitch region where he/she can reach before any other player^8^ and is commonly determined based on Voronoi diagram^7^. The Voronoi tessellations allows the decomposition of a space (e.g., the pitch) into cells (e.g., dominant regions) associated with each point (e.g., players)^9^. This method was applied to determine the players’ dominant region in the football considering the players’ positions and the distance between the players to the nearby players^10^.Then, Filetti, et al.^11^ applied a different Voronoi diagram^12^ that resulted in dominant regions with rounded characteristics. However, the dominant regions determined by Voronoi diagrams^10,11^ are associated with the players because they are the closest^13^, and promote a static perspective that can be representative only of an instant.

Therefore, researchers proposed the use of movement models defined as a set of displacement, velocity, and acceleration data used to represent the players’ movement abilities^8^. Initially, a movement model was created considering the position, displacement, and velocity of the players improving the determination of the dominant region, making it more compatible with human physical capacity^8,14^. Then, an improvement of the movement model was proposed, by adding a resistive force to avoid the velocity increase up to infinity, caused by the acceleration considered as a constant^15,16^. However, the players’ dominant regions like other tactical behaviours in the football (e.g., distance between players, teams effective playing space) can be influenced by several match situations^13^. Thus, a recent study^17^ developed the probabilistic movement model based on the players’ positional data from an official match to determine the dominant regions with even more realism. The probabilistic movement model proposed^17^ considers the players’ position, displacement, and velocity to determine the dominant regions in a time-lag window of one second. Brefeld, et al.^17^ used the velocity discriminated into five different speed intervals (Stand, Walk, Jog, Run, and Sprint), considered the playing positions to generate the movement models and computed the probabilities of the players to reach a given position over time.

The studies using movement models^8,14–17^ can capture the dynamical characteristics of human displacement, elucidating the relevance of the players’ kinematic variables to determine their dominant regions. In the dominant regions, these studies represent the environment of dynamical changes that occur in a football match, previously neglected using the most straightforward Voronoi diagram. However, there is still an overestimation in the players’ dominant region, as Voronoi diagram, because all the pitch regions are associated with a player. For those literature models^8,14–17^, every region of the pitch is dominated by a given player and there are no free spaces, which is a limitation of the proposals presented that can lead to unsuitable interpretations about the playing space analyses. Thus, proposals based on Voronoi illustration attribute huge players area, intuitively unreal for football players. Thus, our purpose was to determine a novel dominant region calculation based on a movement model that considers players’ positions, displacement, velocity, and acceleration vectors obtained from official matches data. The novelty of our approach is that it considers the pitch regions that the player is unable to reach in a specific time interval are considered free spaces and not as their dominant region. Our proposal promotes information about players dominant regions even more compatible with human physical capacity and makes visible the free spaces in the pitch. Furthermore, inspired by Taylor's Formula of order two, this article improves previous approaches by considering the statistics of the second order of the kinematics variables, namely, the acceleration. We believe that displacements with different accelerations, such as positive and negative accelerations, cannot fit into the same movement model, then in our approach several movement models would be generated.

**Figure 1:**
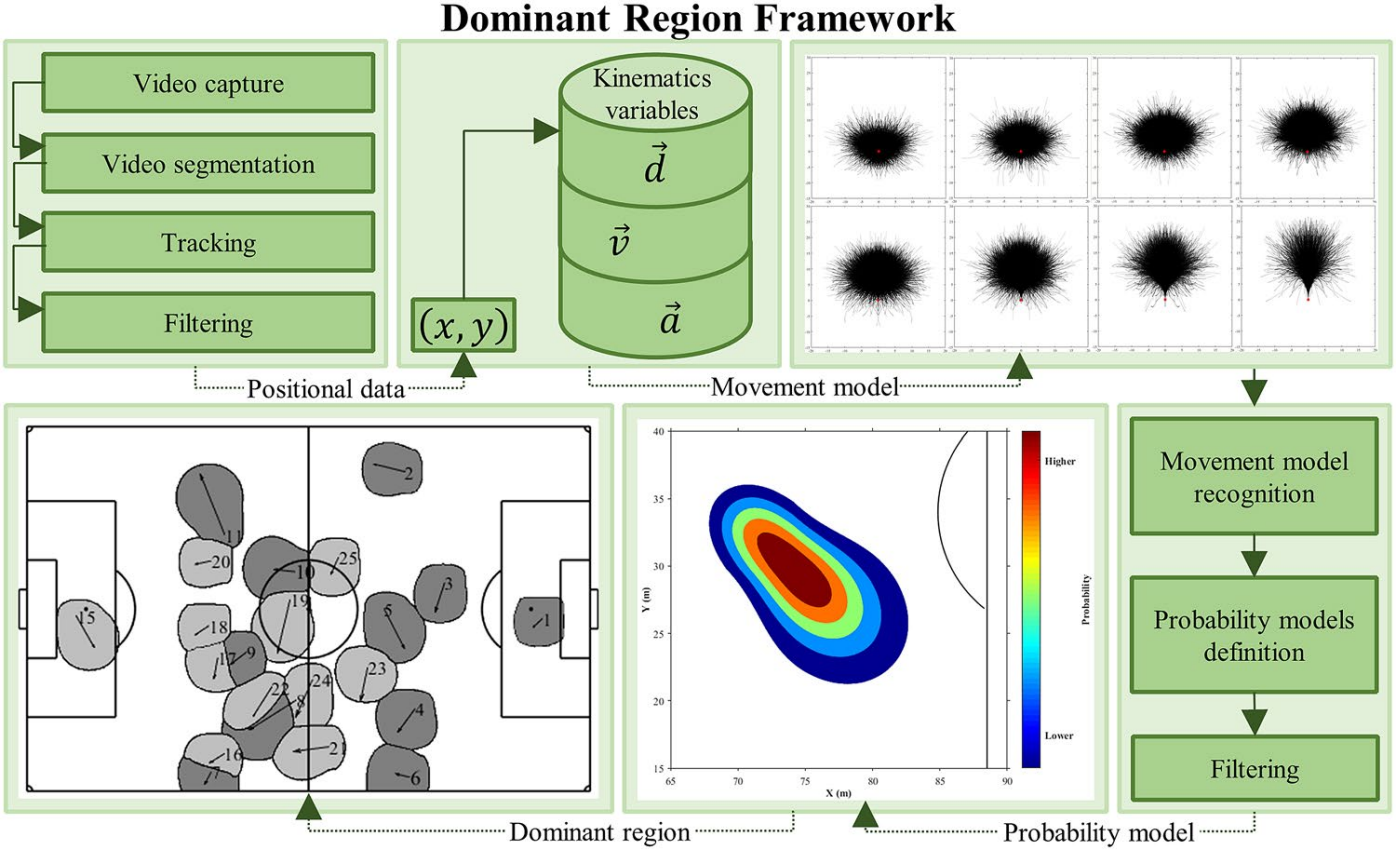
Representation of dominant region framework of our approach, from positional data collection, movement models creation, probability models definition, until to players dominant region determination.

Thus, our proposal is grounded by probabilities models with real instantaneous kinematic variables as input to determine the dominant regions (Fig. 1). In addition, we evaluated the accuracy in determining the players’ dominant regions in different time-lag windows and computed areas of dominant regions and free spaces. Finally, we compared the area of the dominant regions determined by our model and Voronoi diagram. Our initial hypotheses were that (1) the greatest accuracy would be presented in the smallest time-lag window compared to bigger ones; (2) the area of the dominant regions determined through our model would be more realistic to represent the match dynamics and smaller than the dominant regions determined by the Voronoi diagram.

## Methods

### Participants

A total of 109 Brazilian male professional football players was analysed in this study during four official matches of the 2014 Serie A2 of the São Paulo State league resulting in 15,112,163 positional data samples. The matches occurred in non-congested periods (at least 72 h after the previous match), with temperature from 23.2 up to 35.2 °C, relative humidity from 34 up to 86% (according with the National Institute of Meteorology—INMET—Brazil). All matches were performed in accordance with Federation Internationale de Football Association (FIFA) rules, in official stadiums (natural grass, ~ 105 × 68 m). This study was conducted in agreement with ethical recommendations of the Declaration of Helsinki, informed consent was obtained for players data collection and was approved by the Research Ethics Committee of the University of Campinas of Medical Sciences (Protocol #299797418.2.0000.5404).

### Data collection

Four digital video cameras recorded the matches’ images at a resolution of 640 × 480 pixels and an acquisition frequency of 30 Hz. The cameras remained fixed throughout the match at the highest points of the stadiums. After the matches, the video sequences were transferred to a computer and synchronised by identifying specific events in an overlapping region between the cameras.

The players’ positional data during the matches were obtained by videogrammetry (DVideo, v6.02, Campinas, Brazil, www.fef.unicamp.br/fef/laboratorios/lib) using the basic automatic procedures of segmentation, splitting blobs, and tracking^18–21^. The automatic tracking rate of DVideo software was 94% with the remaining processed frames solved manually by an experienced operator. The average error for determination of the player position and the distance covered was 0.3 m and 1.4%, respectively^18–20^. The maximal estimated error^22^ for velocity and acceleration was 1.4% and 2.8%, respectively. The reference coordinate system associated with the pitch was defined from bidimensional real-world coordinates, in meters, of 36 specific points on the pitch measured by a measuring tape before the matches. The image plane coordinates were determined through identification of the corresponding projections of these points in the image via the interface of DVideo software. The players’ bidimensional coordinates relative to the pitch’s coordinate system were obtained by the homography parameters of the image-object transformation calculated based on the direct linear transformation method^23^. The players’ trajectories were filtered using a third-order Butterworth low-pass with a cut-off frequency of 0.4 Hz defined by spectral and residual analyses, based on a previously proposed protocol^18,24^.

### Data analysis

#### Movement models

The movement models were created based on the vectorial kinematics variables of the players obtained during three matches: the instantaneous displacement angles in relation to the longitudinal axis (θ*d*; Eq. 1), velocities (*v*; Eq. 2), and accelerations (*α*; Eq. 3), derived from the players’ positions as a function of time.

1 $${\theta }_{d(i)}={\mathrm{tan}}^{-1}\left(\frac{{y}_{(i+1)}-{y}_{(i)}}{{x}_{(i+1)}-{x}_{(i)}}\right),i=1,\dots ,n-1$$

2 $${v}_{(i)}=\sqrt{{\left(\frac{{x}_{(i+1)}-{x}_{i-1}}{{t}_{(i+1)}-{t}_{(i-1)}}\right)}^{2}+{\left(\frac{{y}_{(i+1)}-{y}_{(i-1)}}{{t}_{(i+1)}-{t}_{(i-1)}}\right)}^{2}},i=2,\dots ,n-1$$

3 $${a}_{(i)}=\frac{{v}_{(i+1)}-{v}_{(i-1)}}{{t}_{(i+1)}-{t}_{(i-1)}},i=2,\dots ,n-1$$

where *x* and *y* are the bidimensional coordinates, *i* indicates the instant of time, *n* is the total number of frames, and *t* is the timestamp, in seconds.

We identified the position, displacement, velocity, and acceleration at each instant of time and registered the positions at the future points up to three seconds. The time window for the future positions was chosen based on an experiment with the notational dataset of three matches resulting 2948 passes analysed. This experiment was based on the players’ behaviour when performing passing actions. The player in possession of the ball perceives the environment (e.g., the displacements of other players) and sees the opportunity to pass the ball to a teammate. This phenomenon is described as affordance^2,3^. The players try to perform a pass to a region of control to their teammate, commonly to where he/she is moving toward. Players estimate the region that their teammate can reach in a future time intuitively, a behaviour already described by motor control researches in the interception tasks^25^. Only the successful passes were analysed, i.e., passes that reached the teammate, to ensure that situations which the players have the intention to intercept the ball or clearance actions are excluded from analysis. We identified the moment that the player performed the pass and the moment that his teammate received the ball. Next, we computed the time window between these instants for all passes. Finally, the distribution of these time windows was analysed, and the maximal future time window of three seconds was determined, which represented roughly 95% of the data. (n = 2948; 25th percentile = 1.03 s; 50th percentile = 1.40 s; 75th percentile = 1.86 s; 95th percentile = 3.16 s).

**Figure 2:**
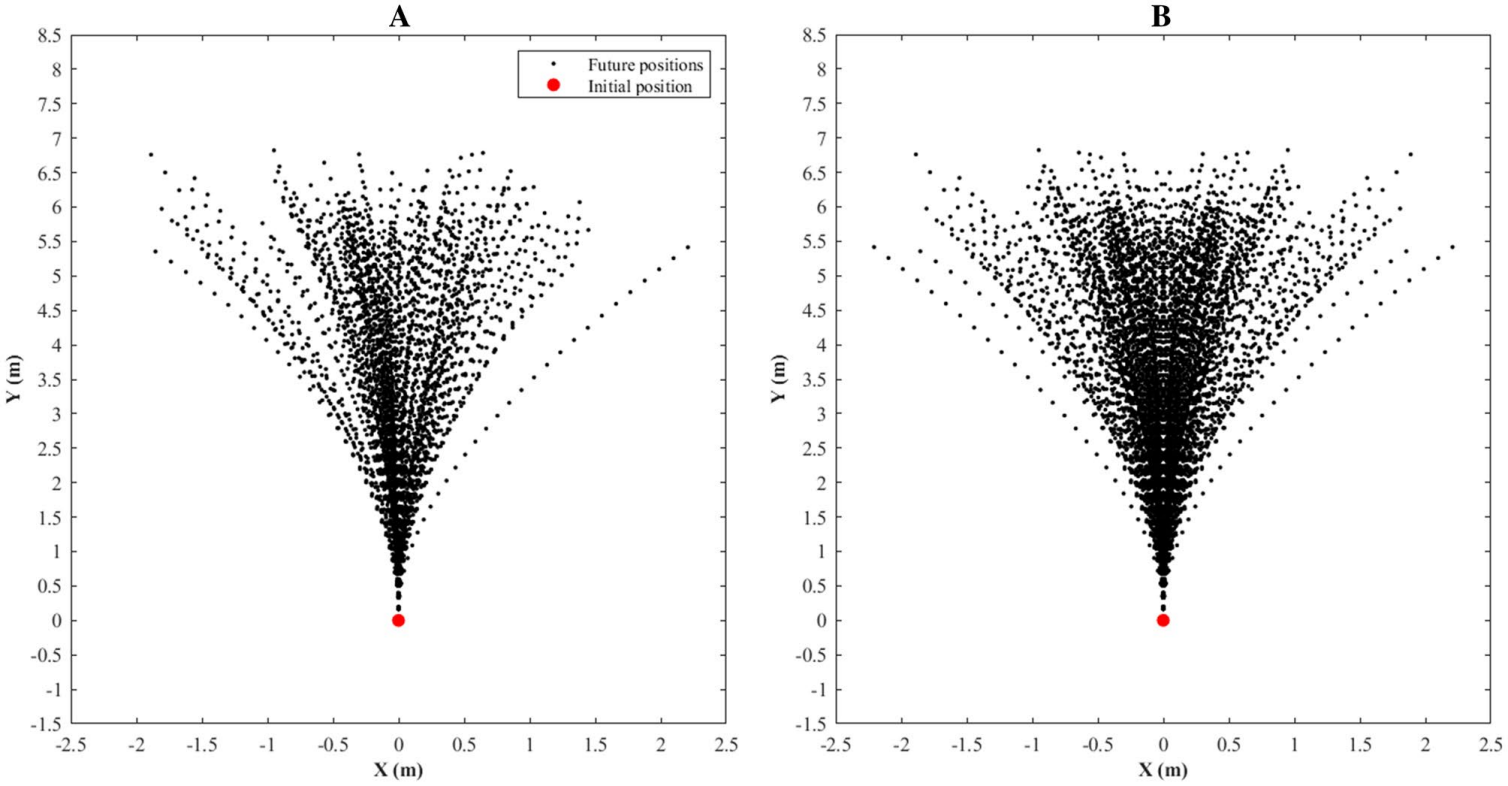
Example of displacements with velocities from 5 to 6 m/s, acceleration from 2 to 3 m/s^2^, and angles rotated 90 degrees and translated to origin in 0 at x and y axis future positions at one-second time window generating a movement model **(A)** and the same movement model after mirroring process **(B)**.

The displacement angles were rotated 90 degrees, translated to origin in 0 at x and y axis (θ*r* and *fp*; Eqs. 4 and 5), and the displacements were mirrored (Fig. 2) around the y axis to balance the curvatures in both sides (*mfp*; Eqs. 6).

4 $${\theta }_{r}=\left\{\begin{array}{c}90+abs\left({\theta }_{d}\right),\quad {\theta }_{d}<0\\ 90-{\theta }_{d},\quad {\theta }_{d}\ge 0\end{array}\right.$$

5 $${fp}_{(i)}=\left[\begin{array}{cc}\mathrm{cos}{\theta }_{r}& -\mathrm{sin}{\theta }_{r}\\ \mathrm{sin}{\theta }_{r}& \mathrm{cos}{\theta }_{r}\end{array}\right]\times \left[\begin{array}{c}{x}_{(i)}-{x}_{1}\\ {y}_{(i)}-{y}_{1}\end{array}\right],i=1,\dots ,90$$

where the *x* and *y* indicate the bidimensional coordinates, *i* refers to an instant of time, and *x*_1_ and *y*_1_ are the initial bidimensional coordinates.

6 $$mfp=\left[\begin{array}{cc}{fp}_{x}& {fp}_{y}\\ {fp}_{x}{^{\prime}}& {fp}_{y}\end{array}\right]$$

**Figure 3:**
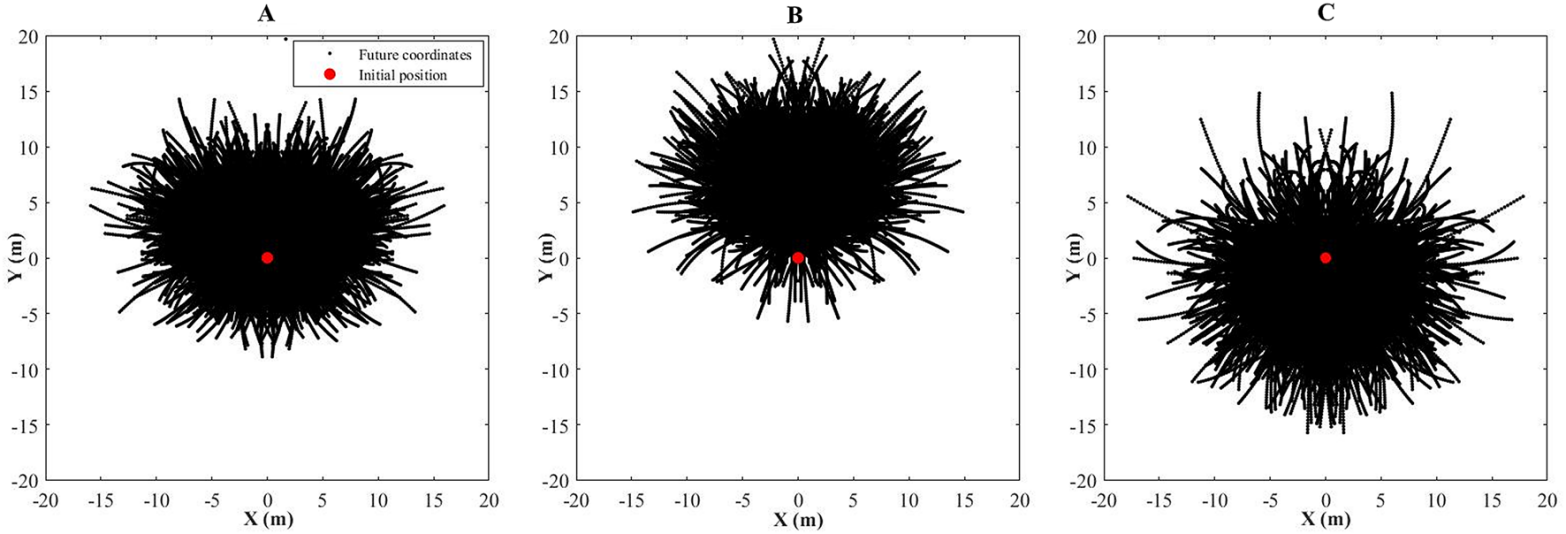
Example of displacements with velocities of 1 m/s at initial instant and future positions at three-seconds time window with accelerations from 0 to 1 m/s^2^ **(A)**, from 3 to 4 m/s^2^ **(B)**, and negative accelerations from 3 to 4 m/s^2^ **(C)**.

**Figure 4:**
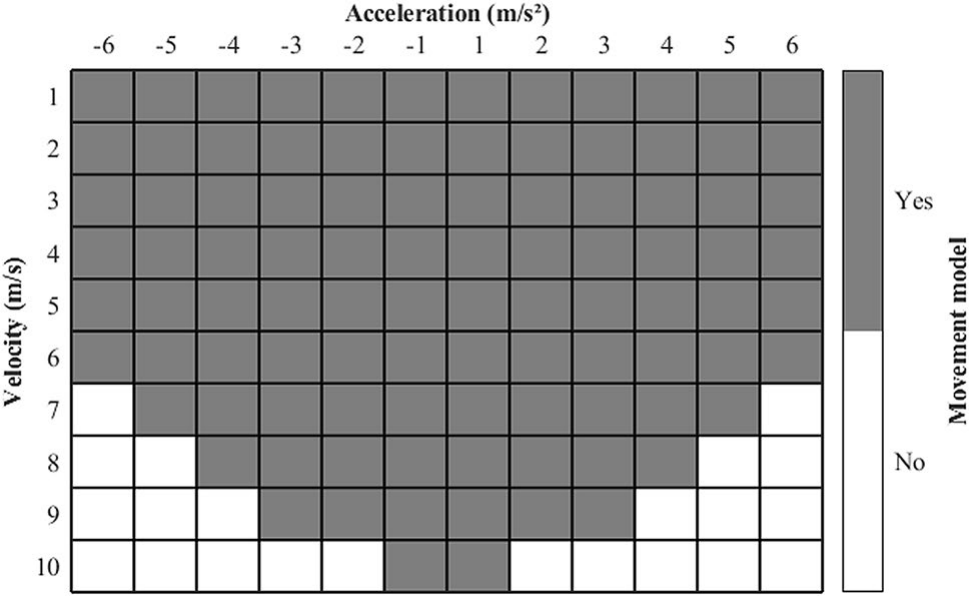
Matrix chart representation of the velocity and acceleration data samples that were used to create their specific movement models according to each range, the combination of velocity and acceleration ranges that generated a movement model are indicated by the gray cells.

The players’ positions considering different time-lags were stored in a matrix, i.e., each cell of this matrix is a movement model (Fig. 3) that correspond the specific combination of velocity (0 up to 10 m/s) and acceleration ranges (− 6 up to 6 m/s^2^) stepping by 1 m/s and 1 m/s^2^ (Fig. 4).

#### Probability models

**Figure 5:**
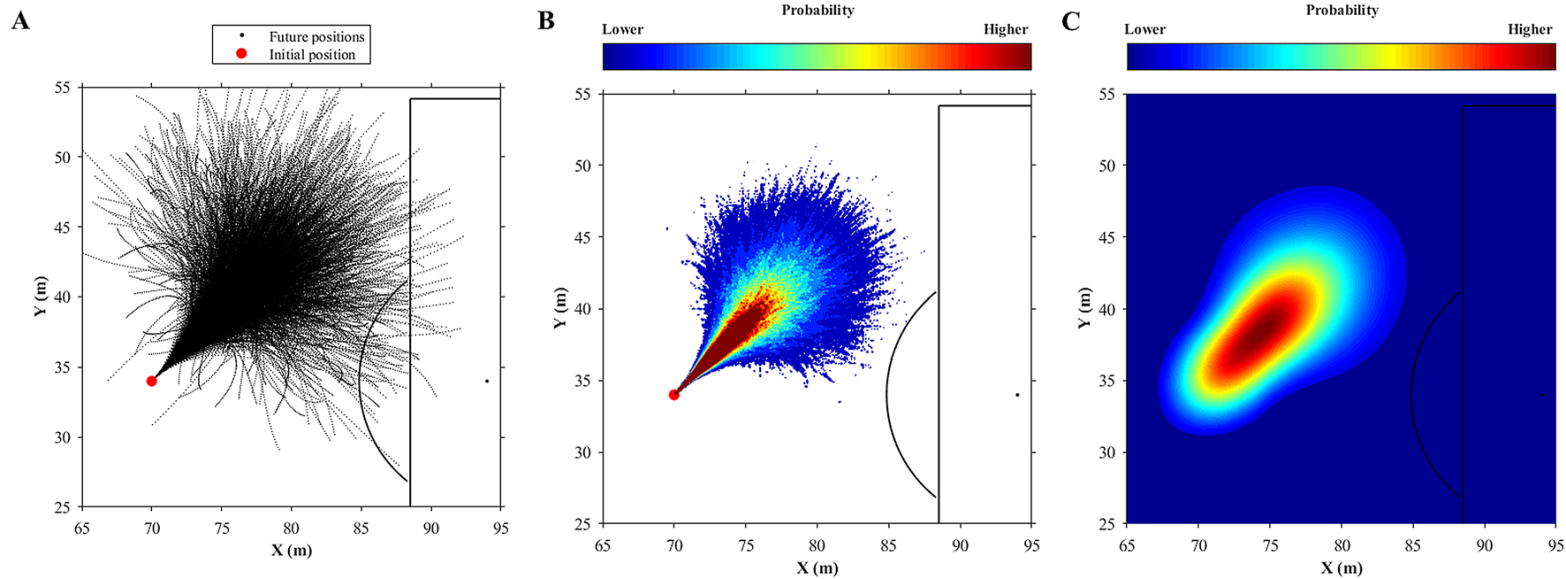
Example of movement model with velocity from 5 to 6 m/s, acceleration from 2 to 3 m/s^2^, and three-seconds future time window rotated and translated to player’s actual displacement at instant time **(A)**, probabilities of the player achieving any region in the pitch calculated from this movement model **(B)**, and probability model after smoothing process **(C)**.

The instantaneous displacement, velocity, and acceleration of players were identified, the respective movement model was recognized, rotated to displacement direction, and translated to actual position of the player (Fig. 5A). The probabilities of a player achieving any region in the pitch were calculated (*pr*; Eq. 7) by histogram function with probability density function normalisation (Fig. 5B).

7 $$pr\left(i,j\right)=\frac{{c}_{(i,j)}}{N\times A},i=1,\dots ,680,j=1,\dots ,1050$$

where *i* and *j* are respectively, the matrix’s rows and columns, *c* indicates the number of elements in the region, *N* is the total number of elements, and *A* is the area of the region.

The grid used to determinate the probabilities range to 0 up to 105 m (x) and 0 up to 68 (y) with a resolution of 0.1 m, generated a matrix (680 × 1050) in which each cell corresponds to probability value of specific region (0.01 m^2^) of the pitch. The matrix’s probability values were smoothed (Fig. 5C) using a 2-D Gaussian filter to reduce the discrete characteristics of the histogram results, improving the spatial coherence, similar to previously applied by Geerts, et al.^26^. The standard deviation (sigma = 20) of the filter was chosen by analyses of the results obtained for all the movement models with different sigma values, to ponder the region probability value from neighbouring regions, preserving the edges and boundaries. The probability models have values ranging from 0 up to 1. Values below 0.001 were rounded to 0 to avoid an association with the very low probability regions with the players’ dominant regions.

#### Dominant regions

The dominant regions in each timestamp were determined by comparing the probability matrices of all players in each instant of time. Thus, each cell of the matrices was compared and registered to the player with greater probability value, resulting in a matrix representing the pitch with the player’s identifier as dominant of each region. The regions with probability values equal to 0 for all players were not associated with any player, and these non-associated regions were defined as free-spaces regions.

The accuracy on determination of the dominant regions was tested for a new dataset: 22 players during the first half of a match (1,856,030 position data samples) that was not used to create the movement models. We measured the accuracy by comparing the dominant region determined for the player at instant time (0.033 s) and the real position of this player at one (T1), two (T2), and three (T3) seconds lag. When the real positions coincided with a region associated with each player, we computed as a correct prediction and the relative frequencies of corrects predictions were calculated. We calculated the areas of the dominant regions and free-spaces with three seconds time-lag in the same dataset used for the accuracy test. The sum of the regions associated with each player resulted in each player dominant region area, as well as the sum of the non-associated regions resulted in the area values of the free-spaces regions.

#### Voronoi regions

The Voronoi diagram was previously proposed by Kim^27^ to determine players' dominant regions. Using the players’ bidimensional coordinates, the Voronoi polygons were defined containing the pitch regions that are closest to each player compared to anyone. The areas of the polygons were calculated to obtain the players’ dominant regions (Voronoi regions) at each instant of time. These procedures were performed using the same dataset that we calculate the areas of the dominant regions determined by our model to enable the comparison between the approaches.

### Statistical analysis

The normality of data distribution was verified by Lilliefors test, then parametric or non-parametric statistics methods are applied accordantly. The percentage of correct predictions are presented in mean and standard deviation, and the dominant region areas are presented in median and interquartile range. Furthermore, we calculated the 95% confidence interval (CI) with lower limit (LL) and upper limit (UP) of means and medians. The Wilcoxon Rank Sum Test was used to compare the players’ dominant regions determined by our proposed model and players’ Voronoi regions, followed by the calculation of the effect size (r) according to proposed by Rosenthal^28^ and classifications were adopted according to the proposal of Cohen^29^. The statistical significance was set as P < 0.05.

## Results

**Figure 6:**
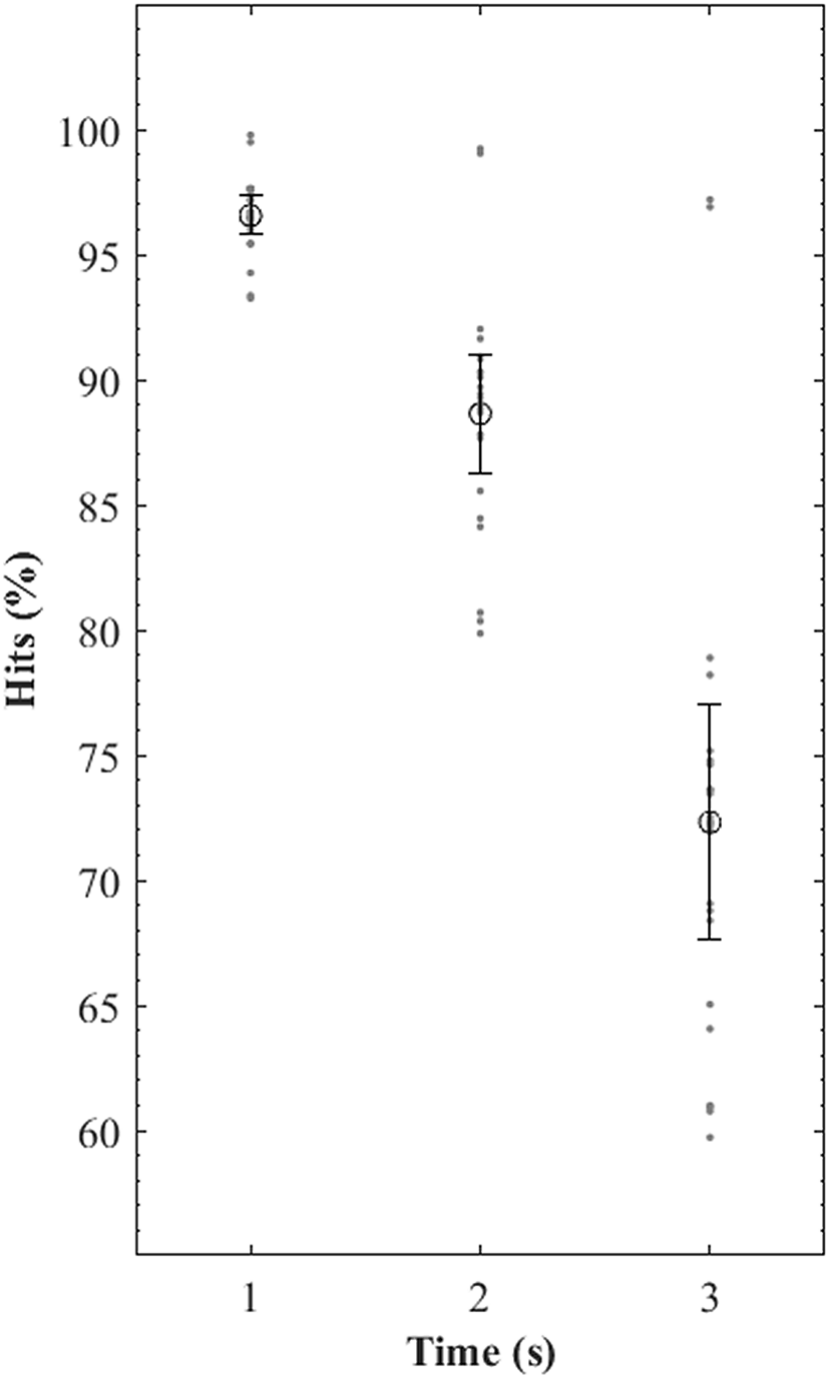
Representation of means (circles), standard deviations (bars), and individual values (dots) of percentage hits in determining players’ dominant regions (future positions coincided with a region associated with them) at different future time windows (one-, two-, and three-seconds lag).

As reported before, the correct predictions were computed when the players’ real future positions coincided with a region associated with them. The means, standard deviations, and individual values of percentage of correct predictions for T1, T2, and T3 for all players are presented in Fig. 6. The greater values of correct predictions were obtained for the lower time window. The mean percentage of correct prediction found for T1 was 96.56% (95% CI: 0.62; LL: 95.94; UL: 97.17), for T2 88.64% (95% CI: 1.90; LL: 86.74; UL: 90.54), and for T3 72.31% (95% CI: 3.76; LL: 68.55; UL: 76.07).

**Figure 7:**
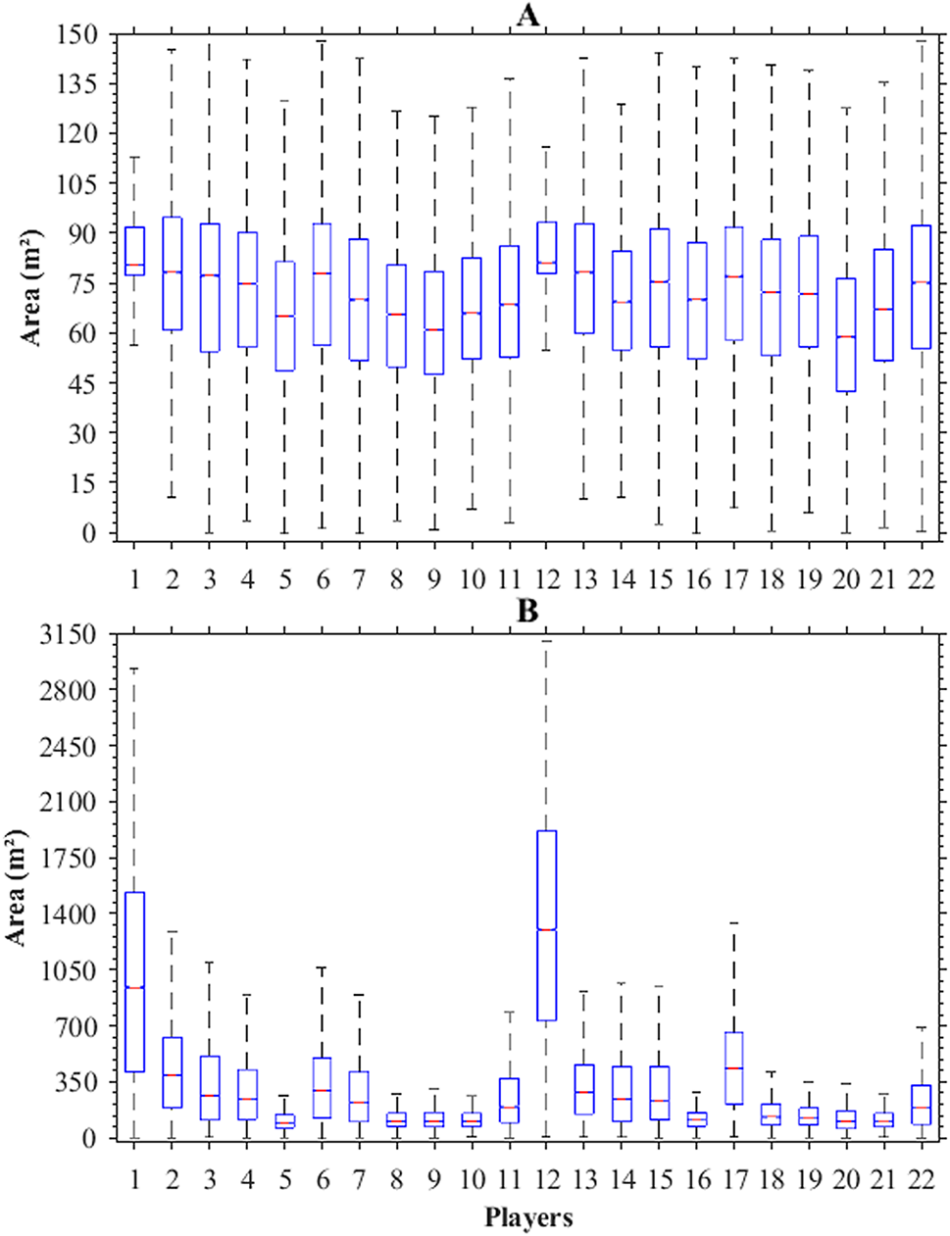
Boxplots represent median (red bars), 25th and 75th percentile (blue boxes), minimum and maximum (black bars) of the dominant region areas (m^2^) determined by our movement model **(A)** and Voronoi regions **(B)** of each player.

Figure 7 presents boxplots representing the area of dominant regions determined by our movement model (Fig. 7A) and Voronoi regions (Fig. 7B) of each player. The experimental results showed that the players’ dominant regions areas determined by our movement model are lower than Voronoi regions (*P* < 0.01; r = 0.53; Medium). The median area of the players’ dominant regions determined by our movement model was 73 m^2^ (95% CI: 0.04; LL: 73.09; UL: 73.17) and interquartile range of 35 m^2^ (P25%: 54; P75%: 89). The median area of the players’ Voronoi regions was 171 m^2^ (95% CI: 0.37; LL: 170.74; UL: 171.49) with the interquartile range of 324 m^2^ (P25%: 88; P75%: 411).

**Figure 8:**
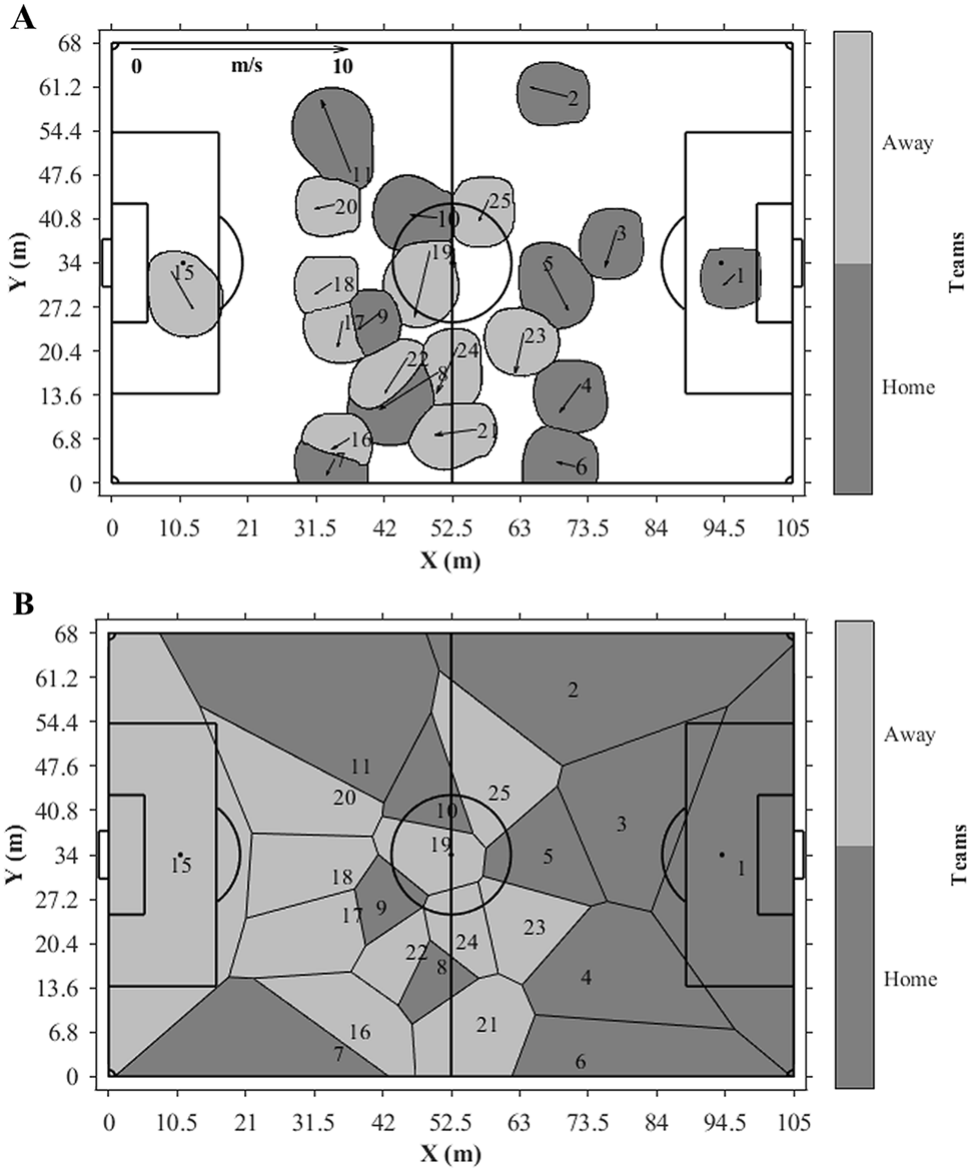
Example of the dominant regions for all players of the two teams determined at same instant time by our movement model with three-seconds future time window **(A)** and Voronoi regions **(B)**, initial positions are represented by the numbers and displacement directions by the vectors.

Additionally, Fig. 8 shows an example of the dominant regions for all players determined at a given instant time by our movement model (Fig. 8A) and using the Voronoi diagram (Fig. 8B). Figure 8A makes it possible to visualize the free-spaces regions generated by our movement model. The area of these regions was computed, and the median area of free spaces was 5,537 m^2^ (95% CI: 1.85; LL: 5,535.11; UL: 5,538.81) and interquartile range of 342 m^2^ (P25%: 5,371; P75%: 5,712), with minimum of 4,677 m^2^ and maximum of 6,476 m^2^. Intuitively, from the model proposed in the present study, it is possible to visualize a more realistic space sharing among the players considering football as an invasion sport.

## Discussion

The purpose of this study was to propose a novel model to calculate football players' dominant regions based on players’ kinematics variables during real matches situations and present the area of dominant regions and free spaces on the pitch. We analysed the accuracy in the determining the players’ dominant regions in different time-lag windows (T1, T2, and T3) and compared the area of dominant regions determined by our movement model and Voronoi regions. The main findings demonstrated an overall high accuracy, but lower values for the dominant regions determined at T3 time-lag. Additionally, Voronoi regions presented greater area values. These results corroborate our initial hypothesis that suggested greater accuracy in the smallest future time windows compared to the largest and the smallest area values of the dominant regions determined using the proposed movement model than the Voronoi regions.

The accuracy of the players’ dominant regions determination enables to verify if the players were in the regions predicted. The main findings showed an expected reduction of accuracy when the time-lag window increases because predicting events that occur in the shortest time can be more precise. This behaviour possibly occurs due the dynamic nature of football, in which the actions of one player are influenced by the movements of the other players or balls events^30^. Similarly, another study that analysed the elite South American players’ work-rate profiles during international football matches reported changes in activities approximately every four seconds^31^. Even though the lowest mean value of accuracy achieved was 72.31% for T3, which is a promising result. It was not possible to draw a parallel with related studies found in the literature because the authors did not report the accuracy of each method. Furthermore, there is no consensus regarding the time windows to create the movement models, varying from two seconds^15,16^, one second^17^, and the shortest time to achieve a specific point^8,14^.

When we analysed the players’ dominant regions areas, the main finding was that the experiment showed a higher median area value for Voronoi regions compared to dominant regions determined by our movement model. Only the study performed by Ueda, et al.^16^ presented dominant regions’ area values, however, the authors analysed the teams’ dominant regions instead of the players. On the other hand, the statistical significance and the effect size presented in our experiment suggest that the same behaviour may be found in further experiments. The lowest areas of dominant regions determined by our movement model are probably because this model considers regions labelled as free space, which does not occur in the Voronoi regions (Fig. 8B). Our study included this concept to avoid that the regions without clear dominance were associated as players’ dominant regions. For instance, during a corner kick situation, the Voronoi regions consider that defenders of the offensive team have dominance of their defensive pitch once they are the closest players, but a fast attack after the corner kick may show that this controlled area is not real because players with greater velocity and acceleration may reach it after few seconds. That is the novelty of our model, showing that the defenders are not really “controlling” these regions that are associated with them following the Voronoi concept. It is important to highlight that the median area value of regions without dominance corresponds to approximately 77% of the pitch’s area, with a minimum close to 66% and maximum of 91%. This maximum value probably occurred during specific events of the matches, e.g., during a corner kick, almost all players share a small region of the pitch, with great proximity between opponent players^32^.

Regions without dominance can be considered free spaces that are distant from the game concentration and physically impossible to be reached for a player in a short period, i.e., regions that are closer to the ball locations generally contain a greater number of players. This behaviour occurs because the ball works as an attractor during a football match^30^. In a similar way, Narizuka, et al.^33^ evaluated the pitch regions, weighting each one in relation to the degree of sparsity and reported that the densest regions are almost located within the teams’ formation, considered as the standard deviation of players’ positions centroid. On the other hand, the regions without dominance may represent key spaces that can be explored by the players to increase the chances of success in offensive attempts or prevent the opponent offensive attempts. A previous study reported that the space dominance on the attacking third of the pitch, computed using Voronoi diagram, was related with the number of goals scored and the probability of the winning the match^34^. Using Voronoi diagram, Ueda, et al.^16^ found that teams presented narrower dominant regions area values in successful offensive performance when the ball possession was acquired near the central region of the pitch. These studies demonstrated the relevance of the team’s dominant regions and their relationship with the performance during football matches^16,34^. Therefore, teams’ spatial dominance assessed by the dominant region analyses can be useful to professionals of sports, making it possible to determine the dominance in important regions of the pitch or during specific situations of the matches. Furthermore, the dominant region by our proposed model can evaluate the teams’ spatial dominance enabling the coaches to know the contribution of each player, directly related to their physical performance during the matches.

The findings of our study should be interpreted considering the future time window used because it influences the results, e.g., a larger time-lag to create the movement models can result in larger players’ dominant regions and smaller free-space regions. This limitation was previously pointed by Rico-Gonzalez, et al.^7^ that suggest caution when analysing dominant region values based on future time windows because the regions that the players reach do not depend exclusively on the physical fitness, but on the time interval used and other factors, such as players’ decisions making that occur during this period. Another relevant point is that studies with different future time windows definitions make the comparison difficult, as mentioned above in this discussion. Thus, can be interesting to standardize the future time window for advances in research with practical application on this topic.

In conclusion, in the present study, we presented a new approach to football players’ dominant regions analysis, based on a movement model created from their real positions, displacement, velocity, and acceleration vectors. The data accuracy demonstrated acceptable values even at larger time-lag windows than the ones reported in the literature. The players’ dominant regions area values computed by our movement model were lower than Voronoi regions. In addition, we showed that the regions without dominance represented a considerable part of the pitch. These findings indicate that the proposed movement model is more realistic representing match dynamics and can be a useful method to evaluate the players dominance during football matches. Coaches can take advantage of the method to analyse opponent teams and to assist their own teams during interventions and planning tactical training.

## Data Availability

The datasets generated during and/or analysed during the current study are available from the corresponding author on reasonable request.
